# Paclitaxel/carboplatin with or without sorafenib in the first-line treatment of patients with stage III/IV epithelial ovarian cancer: a randomized phase II study of the Sarah Cannon Research Institute

**DOI:** 10.1002/cam4.376

**Published:** 2014-12-31

**Authors:** John D Hainsworth, Dana S Thompson, John A Bismayer, Victor G Gian, William M Merritt, Robert C Whorf, Lindsey H Finney, B Stephens Dudley

**Affiliations:** 1 Sarah Cannon Research InstituteNashville, Tennessee; 2 Tennessee Oncology, PLLCNashville, Tennessee; 3 Oncology Hematology CareCincinnati, Ohio; 4 South Carolina Oncology AssociatesColumbia, South Carolina; 5 Florida Cancer SpecialistsFort Myers, Florida

**Keywords:** Carboplatin, first-line therapy, ovarian cancer, paclitaxel, sorafenib

## Abstract

This trial compared the efficacy and toxicity of standard first-line treatment with paclitaxel/carboplatin versus paclitaxel/carboplatin plus sorafenib in patients with advanced ovarian carcinoma. Patients with stage 3 or 4 epithelial ovarian cancer with residual measurable disease or elevated CA-125 levels after maximal surgical cytoreduction were randomized (1:1) to receive treatment with paclitaxel (175 mg/m^2^, 3 h infusion, day 1) and carboplatin (AUC 6.0, IV, day 1) with or without sorafenib 400 mg orally twice daily (PO BID). Patients were reevaluated for response after completing 6 weeks of treatment (two cycles); responding or stable patients received six cycles of paclitaxel/carboplatin. Patients receiving the sorafenib-containing regimen continued sorafenib (400 PO BID) for a total of 52 weeks. Eighty-five patients were randomized and received treatment.Efficacy was similar for patients receiving paclitaxel/carboplatin/sorafenib versus paclitaxel/carboplatin: overall response rates 69% versus 74%; median progression-free survival 15.4 versus 16.3 months; 2 year survival 76% versus 81%. The addition of sorafenib added substantially to the toxicity of the regimen; rash, hand–foot syndrome, mucositis, and hypertension were significantly more common in patients treated with sorafenib. The addition of sorafenib to standard paclitaxel/carboplatin did not improve efficacy and substantially increased toxicity in the first-line treatment of advanced epithelial ovarian cancer. Based on evidence from this study and other completed trials, sorafenib is unlikely to have a role in the treatment of ovarian cancer.

## Introduction

Ovarian cancer is the fourth most common cause of cancer death in women, and is the leading cause of gynecologic cancer death in the United States. Most patients have advanced disease at the time of diagnosis, and are therefore incurable with surgical therapy alone. Systemic chemotherapy, following initial cytoreductive surgery, has markedly improved the treatment of patients with advanced ovarian cancer. At present, the combination of paclitaxel and carboplatin is the most widely used chemotherapy regimen, and produces a median survival of ∼36 months 1,2. Although other cytotoxic agents have activity against ovarian cancer, their addition or substitution in the first-line paclitaxel/carboplatin regimen has failed to further improve results.

Vascular endothelial growth factor (VEGF) and angiogenesis are important promoters of progression of ovarian cancer and other cancer types 3. High VEGF levels are associated with advanced disease, as well as decreased overall survival 4,5. Bevacizumab, an antibody inhibiting VEGF, showed single-agent activity against refractory ovarian cancer, and more recently has improved the progression-free survival (PFS) when added to standard chemotherapy 6–8.

Sorafenib is an oral multitargeted tyrosine kinase inhibitor with effects on tumor angiogenesis through inhibition of the VEGF receptor 9. In addition, sorafenib has inhibitory effects on portions of the RAS/RAF/MEK/ERK signaling pathway, which is also frequently activated in advanced ovarian cancer. Previous experience with angiogenesis agents has suggested better efficacy when used in conjunction with chemotherapeutic agents, rather than as single agents. For these reasons, we added sorafenib to a standard paclitaxel/carboplatin regimen, and compared the efficacy and toxicity to a standard paclitaxel/carboplatin regimen in the first-line treatment of patients with advanced ovarian cancer.

## Patients and Methods

This randomized, multicenter, community-based Phase II trial was initiated in January 2007. Fourteen sites in the Sarah Cannon Oncology Research Consortium participated in the trial. Before patients were enrolled, the trial was approved by the Institutional Review Boards of all participating sites. Informed consent was obtained from all patients.

### Eligibility

Eligible patients were women with histologically confirmed stage III or IV epithelial ovarian carcinoma, previously untreated with chemotherapy or radiation therapy. Initial cytoreductive surgery was required. Following surgery, patients were required to have no remaining tumor nodules >3 cm, no residual tumor involvement of the bowel, and no intestinal obstruction. Patients were required to have measurable disease (RECIST) or evaluable disease (no measurable disease with elevated CA-125 level after surgery). Patients with known residual intra-abdominal tumor after cytoreductive surgery who had normal postoperative CT scans and normal CA-125 were ineligible, unless second-look laparotomy was planned for restaging. Additional eligibility requirements included: ECOG (Eastern Cooperative Oncology Group) performance status 0 or 1; adequate blood counts (ANC ≥1500/*μ*L, platelets ≥100,000/*μ*L, hemoglobin ≥9.0 g/dL); adequate liver function (total bilirubin ≤1.5× upper limits of normal [ULN], ALT (alanine aminotransferase) and AST (aspartate aminotransferase) ≤2.5× ULN, or ≤5× ULN if liver metastases present); serum creatinine ≤1.5× ULN.

Patients were excluded for the following reasons: active cardiac disease during the previous 6 months; central nervous system metastases; uncontrolled hypertension; known infection with HIV, hepatitis B, or hepatitis C; major surgery within 4 weeks; any condition affecting the ability to swallow or absorb oral medication. In addition, standard exclusion criteria for patients receiving antiangiogenesis treatment applied in this study.

Prior to entering this study, all patients were required to give written informed consent.

### Pretreatment evaluation

Prior to beginning therapy, all patients had the following evaluations: complete medical history, physical examination, complete blood counts, chemistry profile, prothrombin time, partial thromboplastin time, urinalysis, and serum CA-125 level. Women of childbearing potential were required to have a negative serum pregnancy test. Radiologic evaluation included computed tomography of the chest, abdomen, and pelvis. A CT or MRI scan of the head was also required if suggestive symptoms were present. Tumor measurements were performed in all patients before beginning therapy.

### Treatment

Patients were randomized (1:1) to receive either paclitaxel/carboplatin plus sorafenib or paclitaxel/carboplatin alone. The doses of paclitaxel and carboplatin were identical in both groups: paclitaxel 175 mg/m^2^, 1–3 h IV infusion, day 1, and carboplatin AUC 6.0, 20 min IV infusion, day 1. Patients in Arm A also received sorafenib 400 mg PO BID. Treatment cycles were repeated every 21 days, for a maximum of six cycles.

Patients received standard premedications prior to each dose of paclitaxel, including diphenhydramine 50 mg, cimetidine 300 mg (or equivalent), and dexamethasone 20 mg, all administered intravenously. All patients received prophylactic antiemetics following standard guidelines for moderately emetogenic chemotherapy. Carboplatin AUC dosing was calculated using the Calvert formula 10. Sorafenib was taken orally, twice daily, either without food or with a moderate fat meal. Strong inducers of CYP3A4 enzymes were avoided during sorafenib administration.

After completing two cycles of therapy, patients were reevaluated with repeat CT scans, CA-125 levels, and tumor measurements. Patients with objective response or stable disease continued treatment, and were again reevaluated after completion of therapy (six cycles). Patients in Arm A (sorafenib arm) who continued to have objective response or stable disease after completing six cycles of treatment continued single-agent sorafenib (400 mg PO BID) to complete a total of 12 months, while patients in Arm B were followed without further treatment until tumor progression was documented.

### Dose modifications

Dose modifications for chemotherapy-induced myelosuppression were specified in the protocol. Dose reductions were based on the blood counts determined on the day of scheduled treatment. If the leukocyte count was >3000/*μ*L and the platelet count was >100,000/*μ*L, full doses of all agents were administered. If the leukocyte count was 2000–3000/*μ*L or the platelet count was 75,000–100,000/*μ*L, 75% doses of paclitaxel and carboplatin were given, with the sorafenib dosing unchanged. If the leukocyte count was <2000/*μ*L, or the platelet count was <75,000/*μ*L, administration of paclitaxel and carboplatin was delayed 1 week, or until the leukocyte count had returned to >3000/*μ*L and the platelets were >100,000/*μ*L; at that time, 75% doses of paclitaxel and carboplatin were administered. Sorafenib dosing was interrupted during the delay in administering the chemotherapeutic agents, and then resumed at full dose when the next cycle of paclitaxel and carboplatin was given. All patients who experienced an episode of febrile neutropenia received 75% dosing of paclitaxel and carboplatin during subsequent cycles. Granulocyte colony-stimulating factors were not administered during cycle 1, but thereafter could be used at the discretion of the treating physician.

For patients who developed grade 3 or 4 nonhematologic toxicity considered to be related to paclitaxel and/or carboplatin, dosing was delayed until the toxicity had decreased to grade 1 or less, and then 75% dosing of the offending agent was continued. Patients were removed from the study if grade 3 or 4 nonhematologic toxicity had not improved to grade 1 or less after a 3-week treatment delay.

Dose reductions of sorafenib were specified for expected toxicities including skin rash, hand–foot skin reaction, and hypertension. Sorafenib dosing was interrupted for patients developing grade 3 toxicities, supportive measures were instituted, and sorafenib was reintroduced at one dose level reduction when the toxicity had improved to grade 1 or less. Sorafenib was discontinued in patients who developed grade 4 hypertension. Two dose level reductions of sorafenib were allowed (1st reduction: 400 mg daily; 2nd reduction: 400 mg every other day).

### Definition of response

All patients were reevaluated for disease response to treatment after completion of two treatment cycles, and again after completion of six cycles. In patients with measurable disease, responses were determined using RECIST, version 1.0 11. To be categorized as a complete responder, patients were required to have normalization of the CA-125 level in addition to having complete response by RECIST. For patients who began treatment without measurable disease but with elevated CA-125 levels, normalization of CA-125 for at least 4 weeks (and resolution of any CT abnormalities) was required to be categorized as a complete responder. For patients who began treatment with neither measurable disease nor elevated CA-125, a negative second-look laparotomy (no visual tumor, biopsies negative) was required for classification as a complete responder. Progressive disease was defined according either to RECIST version 1.0 (patients with measurable disease) or by doubling of the nadir CA125 levels in patients with evaluable disease (per Gynecologic Cancer Intergroup criteria 12).

### Statistical considerations

This randomized, phase II trial was designed to compare the efficacy of a novel regimen containing paclitaxel/carboplatin + sorafenib to standard paclitaxel/carboplatin in the first-line treatment of patients with stage III/IV ovarian cancer. The primary endpoint was the proportion of patients with PFS at 2 years. With standard paclitaxel/carboplatin treatment, the subgroup of ovarian cancer patients targeted in this trial has an expected median PFS of ∼20 months, with ∼45% of patients progression free at 2 years 1. We hypothesized that the addition of sorafenib would improve the 2-year PFS rate from 45% to 67.5%. In order to demonstrate this difference, with an 80% power and alpha = 0.10 (1-sided test), patients were randomized 1:1, with a total of 60 patients treated with the investigational regimen, and 60 patients treated with paclitaxel/carboplatin. Although initially designed to accrue 120 patients, this trial was stopped after 85 patients due to slow accrual.

PFS was defined as the interval from the date of study entry to the date of tumor progression or death. Overall survival was measured from the date of study entry until the date of death. Survival curves were constructed using the method of Kaplan and Meier 13. Toxicity was evaluated in all patients who received at least 1 dose of therapy. Toxicity was graded according to the National Cancer Institute Common Terminology Criteria for Adverse Events version 3.0.

## Results

### Patient characteristics

Between January 2007 and October 2011, 85 patients were enrolled onto this clinical trial and randomized to receive either paclitaxel/carboplatin/sorafenib (Arm A; *n* = 43) or paclitaxel/carboplatin (Arm B; *n* = 42). The clinical characteristics of patients in the two treatment groups are summarized in Table1. Most patients had good performance status, stage III ovarian cancer, and intermediate or high-grade tumors. Overall, 39 patients (46%) had measurable lesions following surgical cytoreduction, while 45 patients (53%) had only elevations of CA-125. More patients in Arm A had ECOG performance status 1 (47% vs. 21%; *P* = 0.02), while more patients in Arm B had stage IV disease (33% vs. 21%; *P* = 0.13).

**Table 1:** Patient characteristics

Characteristic	Paclitaxel/carboplatin/sorafenib (*N* = 43)	Paclitaxel/carboplatin (*N* = 42)
Median age, years (range)	63 (31–78)	62 (42–80)
ECOG performance status		
0	23 (53%)	33 (79%)
1	20 (47%)	9 (21%)
Tumor grade		
GX	4 (9%)	5 (12%)
G1	5 (12%)	4 (10%)
G2	5 (12%)	10 (24%)
G3	28 (65%)	23 (55%)
G4	1 (2%)	0
Stage		
III	33 (77%)	28 (67%)
IV	8 (19%)	14 (33%)
Other	2 (5%)	0
Disease status after surgical cytoreduction		
Measurable per RECIST	15 (35%)	24 (57%)
CA 125 elevation only	27 (63%)	18 (43%)
Nonmeasurable, normal CA 125	1 (2%)	0

### Treatment received

**Figure 1:**
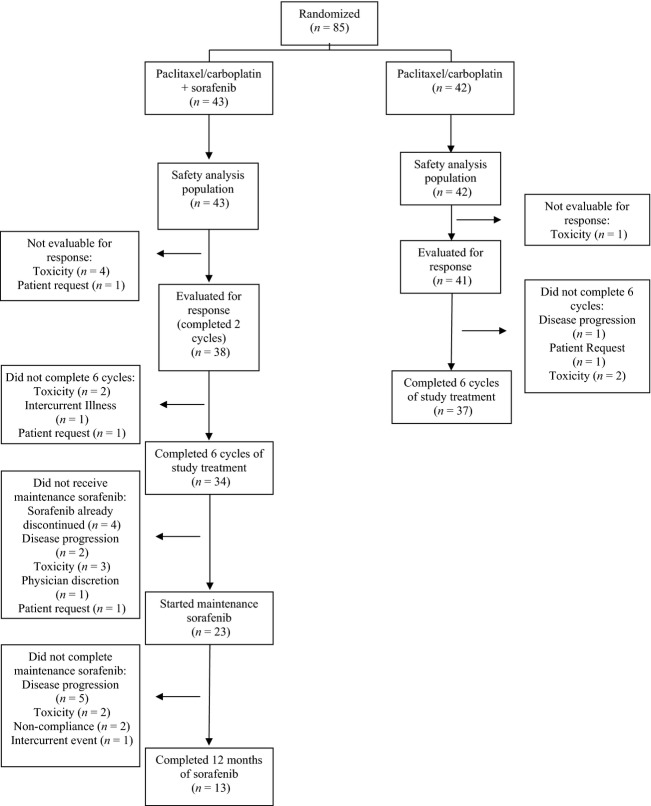
Patient flow diagram.

The patient disposition in this trial is summarized in Figure1. All 85 randomized patients received at least 1 dose of their assigned treatment, and are included in the safety population. Eighty patients (94%) completed two cycles of therapy and were evaluable for response. Seventy-one patients (84%) completed six cycles of paclitaxel/carboplatin (Arm A, 79%; Arm B, 88%). Reasons for not completing six cycles of treatment included treatment-related toxicity (nine patients), patient request (three patients), intercurrent event (one patient), and disease progression (one patient). The percentage of planned paclitaxel and carboplatin received was similar in both treatment arms. Of the patients who completed the paclitaxel/carboplatin dosing, 35 (49%) required dose reductions of paclitaxel and/or carboplatin during their treatment course (Arm A, 18 patients [42%]; Arm B, 17 patients [40%)]).

During the first six cycles of treatment, patients in Arm A receiving sorafenib frequently required dose interruptions or reductions. Four patients discontinued sorafenib, but were able to complete six cycles of paclitaxel/carboplatin. Nineteen patients (44%) required dose reductions of sorafenib. The percentage of the planned sorafenib actually administered during the first six cycles was 62.4%.

Following the six cycles of paclitaxel/carboplatin, 23 patients in Arm A (53%) continued treatment with single-agent sorafenib per protocol. Thirteen patients (30%) completed a total of 12 months of treatment with sorafenib; six of these patients required dose reductions of sorafenib due to toxicity.

### Efficacy

**Figure 2:**
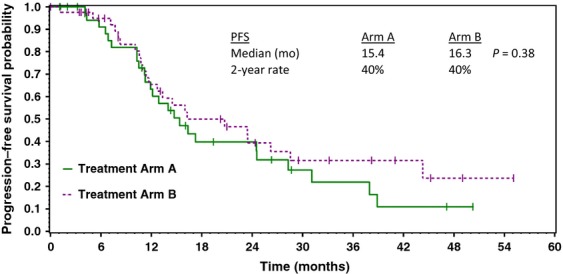
Estimated progression-free survival of patients receiving paclitaxel/carboplatin/sorafenib (Arm A) versus paclitaxel/carboplatin (Arm B).

**Figure 3:**
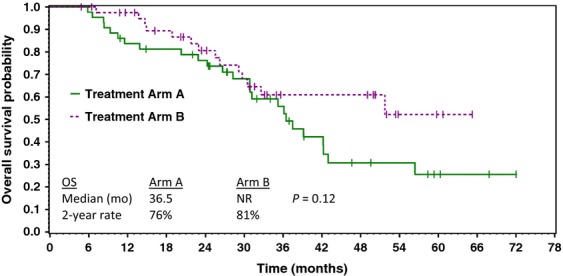
Estimated overall survival of patients receiving paclitaxel/carboplatin/sorafenib (Arm A) versus paclitaxel/carboplatin (Arm B).

The treatment efficacy of the two treatment regimens is compared in Table2. There were no differences in response rate, PFS (Fig.2), or overall survival (Fig.3) between the two regimens. Treatment results with the standard regimen (paclitaxel/carboplatin) were within the range reported in previous studies.

**Table 2:** Treatment efficacy

	Paclitaxel/carboplatin/sorafenib (*N* = 43)	Paclitaxel/carboplatin (*N* = 42)
Response		
Complete	16 (37%)	18 (43%)
Partial	13 (30%)	13 (31%)
Stable	8 (19%)	9 (21%)
Progression	0	1 (2%)
Unevaluable	6 (14%)	1 (2%)
Overall response rate	29/43 (67%)	31/42 (74%)
Progression-free survival		
Median, months	15.4	16.3; *P* = 0.38
2-years (%)	40%	40%
Overall survival		
Median	36.5	NA; *P* = 0.12
2-year	76%	81%
3-year	56%	61%

### Toxicity

Toxicities related to the two treatment regimens administered in this trial are compared in Table3. Hematologic toxicity was similar in both regimens, and was as expected with the paclitaxel/carboplatin combination. Grade 3/4 neutropenia occurred in 26% and 31% of patients, respectively; however, febrile neutropenia was uncommon.

**Table 3:** Treatment-related toxicity

Toxicity	Paclitaxel/carboplatin/sorafenib (*N* = 43)		Paclitaxel/carboplatin (*N* = 42)	
	Grade 1/2	Grade 3/4	Grade 1/2	Grade 3/4
Hematologic				
Neutropenia	17 (40%)	11 (26%)	20 (48%)	13 (31%)
Anemia	22 (51%)	7 (16%)	25 (59%)	5 (12%)
Thrombocytopenia	17 (40%)	9 (21%)	22 (52%)	3 (7%)
Febrile neutropenia	2 (5%)	1 (2%)	0	1 (2%)
Nonhematologic				
Nausea/vomiting	30 (70%)	3 (7%)	34 (81%)	3 (7%)
Peripheral neuropathy	25 (58%)	6 (14%)	25 (58%)	3 (7%)
Fatigue	26 (60%)	3 (7%)	29 (69%)	3 (7%)
Skin rash	27 (63%)	14 (33%)	2 (5%)	1 (2%)
Diarrhea	20 (47%)	2 (5%)	7 (17%)	1 (2%)
Pain-muscle	12 (28%)	0	14 (33%)	0
Hypersensitivity reaction (paclitaxel)	2 (5%)	4 (9%)	0	1 (2%)
Mucositis	16 (37%)	0	7 (17%)	0
Constipation	7 (16%)	0	14 (33%)	0
Pain-joint	9 (21%)	0	11 (26%)	0
Anorexia	9 (21%)	0	8 (19%)	0
Hand–foot syndrome	12 (28%)	4 (9%)	0	0
Abdominal pain	6 (14%)	3 (7%)	2 (5%)	3 (7%)
Weakness	7 (16%)	0	6 (14%)	0
Hypertension	8 (19%)	2 (5%)	2 (5%)	0
Dizziness	6 (14%)	0	6 (14%)	0
Fever (no neutropenia)	6 (14%)	0	5 (12%)	0
Dehydration	4 (9%)	1 (2%)	1 (2%)	3 (7%)
Dyspnea	3 (7%)	0	5 (12%)	0
Headache	5 (12%)	0	3 (7%)	0
Edema	3 (7%)	0	2 (5%)	0
Hyponatremia	0	4 (9%)	0	0
Pruritus	3 (7%)	0	1 (2%)	0
Treatment-related hospitalizations	6 (14%)		6 (14%)	
Treatment-related deaths	0		0	

Patients in Arm A had substantially more grade 3/4 nonhematologic toxicity, primarily skin toxicity, hand–foot syndrome, mucositis, and hypertension. All of these toxicities have been consistently reported with sorafenib. Six patients in each treatment group had treatment-related hospitalizations. No treatment-related deaths occurred.

## Discussion

Advanced epithelial ovarian cancer is a moderately chemosensitive malignancy, and survival is substantially improved when platinum/taxane combinations are administered as first-line therapy. However, since the introduction of the taxanes almost 20 years ago, further therapeutic improvements have been difficult. Recently, inhibition of angiogenesis has been validated as a therapeutic strategy in ovarian cancer, and several inhibitors of either VEGF or VEGFR have shown single-agent activity 6,14,15. When combined with chemotherapy in relapsed or refractory ovarian cancer, bevacizumab has significantly extended progression-free survival, although its impact on overall survival is still unclear 7,8,16.

In this randomized phase II trial, patients with previously untreated advanced ovarian cancer received treatment with either paclitaxel/carboplatin or paclitaxel/carboplatin/sorafenib following maximal surgical cytoreduction. Unfortunately, interpretation of the results is hindered since the trial accrued slowly and was closed after 85 of a planned 120 patients had been randomized. However, comparisons of the efficacy of the two regimens showed no differences in overall response rates, PFS, or overall survival. The PFSs for both groups of  patients (paclitaxel/carboplatin/sorafenib, 15.4 months; paclitaxel/carboplatin, 16.3 months) were similar to those previously reported using standard therapy for advanced ovarian cancer. As anticipated, the patients who received sorafenib had more toxicity. The additional toxicity consisted primarily of well described sorafenib-related toxicity including skin toxicity, hand–foot syndrome, mucositis, and hypertension. The difficulty in tolerating the sorafenib-containing regimen resulted in a high rate of sorafenib dose reductions and discontinuations, and may have had an adverse impact on the efficacy of the regimen.

Since this study was initiated, several other trials have explored the potential role of sorafenib in the treatment of ovarian cancer, with generally disappointing results. Most of the experience has been in patients who were relapsed or refractory after previous platinum-based chemotherapy. The Gynecologic Oncology Group enrolled 73 patients with recurrent/refractory ovarian cancer to a trial of single-agent sorafenib 17. In this group of patients, activity was modest (3.4% partial responses, 24% progression-free ≥6 months), and the patients had substantial toxicity. Sorafenib was administered in combination with gemcitabine to a group of 43 patients with recurrent/refractory ovarian cancer 18. Two patients (4.7%) had partial responses (not meeting the primary endpoint of the study); 23% remained progression-free for more than 6 months.

In one small trial, an attempt was made to add sorafenib to combination chemotherapy for ovarian cancer 19. This study was stopped early, because three of the first four patients treated had life-threatening events considered possibly related to treatment (cardiac output failure, myocardial infarction, and anastomotic leak). Two of these patients also had progression of their ovarian cancer during the first four courses of therapy. Although the addition of sorafenib increased toxicity in our trial (using an almost identical regimen), we did not encounter the severe toxicity described in this trial, nor did we experience an unusual number of patients with primary refractory disease.

Finally, a randomized phase II trial compared maintenance therapy with sorafenib versus placebo in patients who had achieved complete remission following standard first-line chemotherapy 20. Two hundred forty-six patients were randomized; there was no difference between the PFS of patients receiving sorafenib versus placebo (median 12.7 vs. 15.7 months; hazard ratio 1.09). More patients receiving sorafenib stopped maintenance therapy early (treatment duration 17.6 vs. 51.9 weeks), and more required dose reductions (67.5% vs. 30.1%).

In contrast to the discouraging findings with sorafenib-containing regimens in ovarian cancer, four phase III trials have demonstrated a prolongation of PFS when bevacizumab is added to chemotherapy 7,8,16,21. These benefits have been consistent whether bevacizumab is administered as part of first-line treatment or in patients with relapsed disease. Most recently, the AURELIA trial demonstrated an improvement in median PFS from 3.4 to 6.7 months when bevacizumab was added to single-agent chemotherapy in the treatment of patients with platinum-resistant ovarian cancer 21.

As more evidence accumulates, it is evident that sorafenib is not the angiogenesis inhibitor-of-choice in the treatment of patients with advanced ovarian cancer. Sorafenib has modest activity as a single-agent in relapsed ovarian cancer, and although not studied in phase III trials, the addition of sorafenib to effective combination chemotherapy regimens has not suggested increased efficacy in any trial. In addition, sorafenib (at the standard dose of 400 mg BID) is tolerated poorly by ovarian cancer patients, even when used as a single agent; difficulties in adding sorafenib to combination chemotherapy have also been demonstrated in other diseases 22,23. It appears unlikely that sorafenib has a role in the treatment of ovarian cancer, and future clinical trials should focus on agents with new targets.

## Conflict of Interest

None declared.
